# Development and validation of the Environmental Confinement Stressors Scale (ECSS-20)

**DOI:** 10.3389/fpsyg.2024.1386235

**Published:** 2024-07-15

**Authors:** J. Francisco Santibáñez-Palma, Rodrigo Ferrer-Urbina, Geraldy Sepúlveda-Páez, Josefa Bravo de la Fuente, Karina Alarcón-Castillo

**Affiliations:** Escuela de Psicología y Filosofía, Universidad de Tarapacá, Arica, Chile

**Keywords:** environmental stressors, COVID-19, confinement, ESEM, psychometric scales development

## Abstract

The COVID-19 pandemic has generated a global crisis with severe consequences for public health. There have been negative impacts on people’s quality of life and mental health due to various stressors arising in this context, such as physical, social, economic, and psychological challenges. Noteworthy among these are the indirect effects of health measures, especially social distancing and confinement, which have significantly altered people’s daily lives and social activities, producing high levels of anxiety, depression, and stress. This study proposes developing and validating a cross-sectional scale called the “Environmental Stressors Scale (ECSS-20)” to address the need to measure the impact of environmental stressors during confinement. The scale, which has been validated following ethical and methodological guidelines, consists of four dimensions: economic stressors (EE), social activities (SA), habitability (H), and exposure to virtual media (EMV). A pilot study (*n* = 113) and a main study (*n* = 314) were applied. The results showed that the instrument has a reliable and valid structure, with satisfactory internal consistency and factorial validity. Likewise, gender invariance tests supported its suitability for its applicability to women and men. Overall, the ECSS-20 is a valuable instrument for assessing the impact of confinement and improving the understanding of people’s subjective experiences in this situation. Future research could further develop its applicability in different contexts and populations to better understand its usefulness and psychometric properties.

## Introduction

The pandemic context resulting from COVID-19 has depicted a global emergency, from the health point of view, with more than 6,987,222 deaths to date [World Health Organization (WHO), 2023], and in terms of the quality of life and mental health of people, due to the set of stressors that arose in this context (i.e., physical, social, economic, and psychological) (Bavel et al., 2020; Ornell et al., 2020; Wang et al., 2020; Wetherall et al., 2022). Within these stressors are those that emerged from the indirect effects of health policies and containment efforts, specifically, the policies of confinement and social distancing (Caqueo-Urízar et al., 2020), designed to reduce personal interactions and movements (Maier and Brockmann, 2020; Mayr et al., 2020; Badenes-Plá, 2022; Yu et al., 2023), which generated changes in the social and daily activities of the population (e.g., studies, work, intimate relationships, financial management, home habitability) (Ammar et al., 2020; Gloster et al., 2020; Marroquín et al., 2020; Akbari et al., 2021; Amerio et al., 2021; Gruber et al., 2021; Lal et al., 2021; Manchia et al., 2022; Quintana, 2022; von Keyserlingk et al., 2022).

As is well known, the social environment systematically influences health, considering social, psychological, economic, demographic, local and cultural aspects (Wild, 2005; McMichael, 2011; Pettini and Mazzocco, 2022; Whipple and Evans, 2022; Eyre et al., 2023; Gudi-Mindermann et al., 2023; Ibanez and Eyre, 2023). In this sense, any variation in these aspects will impact the health status of people, being detrimental or beneficial to the population. For example, there is ample evidence on how contexts of physical and social isolation (i.e., plague, influenza, cholera, leprosy, or others) and subjective social isolation are associated with negative impacts on mental health (Cacioppo and Hawkley, 2009; Cacioppo and Cacioppo, 2014; Cacioppo et al., 2015; Bzdok and Dunbar, 2022; Gilbar et al., 2022). The growth of such literature has been exponential during and following the COVID-19 pandemic (Jakovljevic et al., 2020; Mukhtar, 2020; Wilder-Smith and Freedman, 2020; Clair et al., 2021; Liu et al., 2021; Neville et al., 2021; Takian et al., 2021), highlighting negative psychological impacts (i.e., distress, anxiety, depression, and high levels of stress) that are primarily attributed to side effects of confinement (Cuadra-Martínez et al., 2020; Gloster et al., 2020; Kujawa et al., 2020; Kokkinos et al., 2022; Nanath et al., 2022). These effects, which have been reported in all types of populations (e.g., children, adolescents, adults, pregnant women, senior citizens, among others) (Manchia et al., 2022), would be explained by the increase in environmental stressors and the variability of coping resources (Verdolini et al., 2021; McLaughlin et al., 2022; Tracy et al., 2022; Yang et al., 2022; Delhey et al., 2023). Now, stress refers to an emergent relationship between the person and the environment (Lazarus and Cohen, 1977) involving environmental stimuli, their evaluation and the organism’s response (Cohen et al., 1983, 2007, 2019; Segerstrom and O’Connor, 2012). Likewise, *environmental stress* is defined as the physiological, cognitive, and emotional response that people may experience to various environmental situations, whether at the macro-level (e.g., population density in a city) or in the immediate environment (e.g., housing conditions; Gatersleben and Griffin, 2017).

A wide range of environmental stressors derived from confinement are observed in the literature (Ellen et al., 2021; Hussong et al., 2021; Kumar and Shah, 2021; Kunzler et al., 2021; Salazar et al., 2021; Sheek-Hussein et al., 2021; Szkody et al., 2021; Valdés-Florido et al., 2022; Morgado et al., 2023). Researched are: (1) economic stressors (ES), which refer to the perceived economic impact that a variation in the estimated household budget generates, and thus impacts on job insecurity and economic livelihood in households (Bazzoli et al., 2021; Friedline et al., 2021; Low and Mounts, 2022) (2) everyday activities (EA), understood as the impact on the performance of routine activities, being considered an individual facet of social practice (Rieger and Wang, 2020; Ellen et al., 2021); (3) social activities (SA), which refers to the perceived impact on social recreation activities such as interaction with others and leisure (Kunzler et al., 2021; Nielsen et al., 2021); (4) home habitability (H), referring to the operational housing conditions and comfort (i.e., conditions necessary to satisfy the physical, biological, psychological, and social well-being) of those who inhabit a dwelling (Corral-Verdugo et al., 2011; Zulaica and Oriolani, 2019); and (5) virtual media exposure (VME), understood as the level of person’s exposure to, or interaction with, virtual or technological media (e.g., television viewing, computer use, use of social networks, websites and mobile applications; Dubey, 2020; Rivest-Beauregard et al., 2022).

Consequently, the frequency of environmental stressors, whether higher or lower, defines how confinement or other situations that cause stressful environmental changes in people’s habitability (e.g., population displacement due to natural disasters and/or in search of shelter) will be experienced, thereby affecting their health (Choi et al., 2020; Kim et al., 2021; Bzdok and Dunbar, 2022). Thus, the evaluation of these environmental factors will provide a more complete view of how the health of the population is affected, and how to counteract this situation. In this way, it will be possible to generate preventive actions, either at the individual or collective level, that are aimed at the well-being of people.

In this line, and considering that efforts to study this phenomenon lack a psychometric instrument with evidence of validity, the present study aims to design a scale that assesses the perception of change in environmental conditions of confinement incorporating a cross-sectional approach, and thus reduce the existing gap in research on the measurement of the impact of confinement (Manchia et al., 2022). To this end, updated evidence of both reliability and validity is presented, following the guidelines of ethical and methodological standards recognized in the field of psychometric evaluation (Prieto and Delgado, 2010; American Educational Research Association, 2014; Muñiz and Fonseca-Pedrero, 2019).

## Method

### Procedures

The study was approved by the Ethics Committee of the Universidad de Tarapacá. An instrumental study was conducted, i.e., a battery of instruments was applied, with a cross-sectional design, i.e., applied over a period of time (Ato et al., 2013).

Initially, 68 items were profiled and evaluated by expert judges (two judges with experience in psychometrics and one judge specialized in the health area) in terms of grammatical adequacy (coherence and clarity) and construct representativeness, using a score of “−1, 0, 1” where “1” represents the grammatical adequacy and construct representativeness of the item. Means were then calculated and items with means less than or equal to 0 were eliminated; 45 items were retained from this process and applied to an online pilot study.

The pilot sample was collected through non-probability sampling strategies (Otzen and Manterola, 2017), using snowball and social network strategies (Montero and León, 2007). It consisted of 113 adults between 18 and 51 years of age (M = 27.1; SD = 7.29), 83 women (73.5%), 27 men (23.9%) and 3 (2.7%) individuals who did not identify with any of the aforementioned groups, coming from the Biobío region (45%, *n* = 50), the Arica and Parinacota region (42.3%, *n* = 47) and other regions of the country (12.7%, *n* = 16). It was surveyed online during October 2021 using a Google Form with a response procedure of 30 to 35 min.

Once the pilot sample was collected, an exploratory factor analysis (EFA) was conducted to explore the underlying structure of the data (Costello and Osborne, 2019). In addition, to provide a brief and concise scale representing the construct of interest, items with values below 0.50 on the factor loadings of each item were iteratively removed.

The results of the EFA suggested a new dimension, which included items related to exposure to virtual media (e.g., being exposed to computers or television and participating in virtual meetings). Thus, a 30-item version was obtained, which was applied to the main study sample, which like the pilot sample, was collected through non-probability sampling strategies (Otzen and Manterola, 2017), using snowball and social networking strategies (Montero and León, 2007), during January 2022 using a Google Form with a response procedure of 20 to 25 min.

### Participants

The main study sample consisted of 314 adults between 18 and 79 years of age (M = 27.34; SD = 9.58), 191 women (60.8%), 123 men (39.2%), from the Biobío region (31.5%, *n* = 99), the Arica and Parinacota region (43.8%, *n* = 137), the Metropolitan region (9.6%, *n* = 30), and other regions of the country (15.1%, *n* = 48). The main study was conducted in the classrooms of the Universidad de Tarapacá during April and May 2022, using QR codes and paper-and-pencil surveys.

### Instruments

The *Environmental Confinement Stressors Scale (ECSS-20)* was developed to evaluate the subjective comparison, before and during, of the most predominant environmental stressors established in periods of stress and confinement. The final version of the questionnaire consists of four dimensions of perception: (a) economic stressors (ES), (b) social activities (SA), (c) home habitability (H), and (d) exposure to virtual media (VME), with five items each, for a total of 20 items. The response options have a Likert format of 5 ranked categories (−2 = “Much less than before,” 2 = “Much more than before”). In the EE and EMV dimensions, higher scores are interpreted as experiencing a significant increase in environmental stress than before confinement. In the AS and H dimensions, higher scores are interpreted as experiencing a significant decrease in environmental stress than before confinement. The statements refer to facts and behaviors associated with environmental stressors in confinement.

*Perceived Stress Scale (PSS-14)*: It is a 14 item self-report designed to assess “the degree to which life situations are evaluated as stressful” (Cohen et al., 1983), was applied in the main study. Tapia et al. (2007) validated and adapted this inventory in Chile. In the Chilean population, this inventory has presented a Cronbach’s alpha higher than 0.889 (González-Tovar and Hernández-Rodríguez, 2020). Half of the questions are positively formulated and reverse-coded. Each item is scored on a 5-point scale (0 = never, 4 = very often). Individual scores on the PSS-14 can range from 0 to 56, considering that (1) scores between 0 and 19 would be considered no stress; (2) scores ranging from 2 to 28 are considered low stress; (3) scores ranging from 29 to 38 would be considered moderate stress; and (4) scores ranging from 39 to 56 are considered high perceived stress (Tapia et al., 2007).

### Data analysis

First, in the main investigation, an exploratory structural equation model (ESEM) was performed. An exploratory structural equation model is a statistical modeling technique that combines the advantages of exploratory factor analysis (EFA) and confirmatory factor analysis (CFA), allowing estimating the effects and relationships between variables in a more precise and flexible way by accounting for measurement errors in both dependent and independent variables (Morin et al., 2016; Marsh et al., 2020). Oblimin rotation (Asparouhov and Muthén, 2009) and the weighted least square mean and variance adjusted (WLSMV) estimation method were used for the ESEM, which is robust, with non-normal discrete variables from the matrix of polychoric correlations (Muthén and Asparouhov, 2011; Brown, 2015).

Second, the following cut-off points were considered for the overall model fit: values higher than 0.96 in comparative fit index (CFI) or Tucker–Lewis index (TLI) and values lower than 0.07 in root-mean-squared error of approximation (RMSEA) (Hair et al., 2019). The modification indexes and cross-loadings between various items of different dimensions were analyzed.

Using an iterative approach, three fundamental criteria were applied: selecting items with moderate or vigorous factor loadings (*λ* > 0.50), the elimination of redundant items, and the deletion of items with solid cross-loadings (*λ* > 0.30) (Muthén and Asparouhov, 2012; Xiao et al., 2019).

Third, reliability was estimated for each dimension using Cronbach’s Alpha coefficient. Additionally, McDonald’s hierarchical omega was provided to report more efficient reliability criteria, with values above 0.70 considered acceptable and above 0.80 adequate (Cho and Kim, 2015; McNeish, 2018).

Fourth, to assess the instrument’s stability between different genders (women and men), invariance tests were performed to verify that the scores of the items have the same meaning for both groups and do not present biases (Leitgöb et al., 2023). For this purpose, the increase in RMSEA (> 0.010) and the decrease in CFI (>0.005) were considered as evidence of invariance (Cheung and Rensvold, 2002; Chen, 2007; Dimitrov, 2010).

Finally, to establish existing relationships with other variables, a SET-ESEM was performed between ECSS-20 dimensions and PSS-14 dimensions (Cohen et al., 1983); for this, the WLSMV estimation method and the polychoric correlations matrix were used. Sequential analyses were performed using Mplus (8.0) (Muthén et al., 2017) and Jamovi (2.2.5) (The Jamovi, Project, 2021) statistical software.

## Results

First, based on a qualitative analysis, it was decided to discard one item of the dimension because it loaded positively on the factor despite being classified as an inverse item. Then, a 29 items ESEM model (Model 1; M1) was estimated, as shown in Table 1. This model showed an excellent statistical fit according to the parameters proposed by Hair et al. (2019), for the CFI estimator (CFI = 0.972; TLI = 0.958). However, three items of the everyday activities factor presented relevant cross-loadings (i.e., *λ* > 0.5) on the social activities factor (i.e., perform physical activity; obtain medical or health care easily; take walks/visits).

**Table 1:** Fit indexes for models ESEM and CFA of ECSS-20.

Model	*χ*2	df	*χ*^2^/df	RMSEA	90% CI	CFI	TLI	SRMR
M1	838.537*	271	3.09	0.082	[0.075, 0.088]	0.972	0.958	0.023
M2	730.636*	227	3.21	0.084	[0.077, 0.091]	0.972	0.960	0.024
M3	380.155*	116	3.27	0.085	[0.076, 0.095]	0.977	0.962	0.021
M3a	140.970*	164	0.85	0.069	[0.061, 0.078]	0.978	0.975	0.044

*p < .001. M1, ESEM with four factors, 29 items; M2, ESEM with four factors, 26 items; M3, ESEM with four factors, 20 items; M3a, CFA with four factors, 20 items; *χ*^2^, Chi-square; df, degree of freedom; RMSEA, root mean square error of approximation; 90% CI, confidence interval; CFI, comparative fit index; TLI, Tucker–Lewis index; SRMR, standardized root mean square residual.

Consequently, after a qualitative analysis (i.e., item relevance and construct definition), the items above became part of the social activities factor. Likewise, the remaining items of the everyday activities factor (e.g., carrying out procedures typically) were discarded due to their redundancy, leaving 26 items.

Second, a 4-factor model with 26 items was estimated (Model 2; M2), which presented better internal structure adjustments in the CFI and TLI indicators (CFI = 0.972; TLI = 0.960), compared to M1. As previously mentioned, items with lower factor loadings referring to the factor (*λ* > 0.5) and that presented cross-loadings (*λ* ≤ 0.3) were iteratively eliminated to reduce the number of items in the scale. In total, 20 items were retained, estimated in Model 3 (M3).

The ESEM analysis of M3 presented better internal structure adjustments in the CFI and TLI indicators (CFI = 0.977; TLI = 0.962) than the previous models. This structure was confirmed by performing a CFA, which evidenced a satisfactory fit for the CFI, TLI, and RMSEA indicators (CFI = 0.978; TLI = 0.975; RMSEA = 0.069) (Hair et al., 2019). Finally, the ECSS-20 comprises four dimensions (i.e., ES, SA, H, and VME) and five items per dimension (i.e., 20 items in total).

Table 2 presents the factor loadings with their corresponding factorial covariances and reliability coefficients of the M3. Factorial loadings for this model proved adequate for each factor, and no relevant cross-loadings were observed. Also, structural relationships between dimensions were moderate (*r* > 0.30), mild (*r* > 0.10; Cohen, 1988), and null, and reliability estimates were adequate (*ω* > 0.89; *α* > 0.89; Cho and Kim, 2015).

**Table 2:** Standardized factor loadings resulting from ESEM, factorial covariations and reliability coefficients (Cronbach’s alpha and McDonald’s omega) for each dimension of ECSS-20.

	Descriptive Stadistics			Factor Loadings				Reliability Statistics	
	M (SD)	S	K	ES	SA	H	VME	α if item is dropped	ω if item is dropped
**Economic stressors (ES)**									
1. Difficulties organizing household finances	0.08 (1.15)	−0.263	−0.574	0.804	−0.004	−0.079	0.021	0.888	0.895
2. Difficulties generating income	0.28 (1.10)	−0.315	−0.362	0.762	0.101	−0.077	0.089	0.882	0.890
3. Difficulties covering essential household services	0.04 (0.83)	−0.795	0.334	0.925	−0.048	0.074	−0.026	0.873	0.876
4. Difficulties meeting bank or retail debts	0.08 (1.00)	−0.117	0.081	0.910	0.005	0.032	−0.024	0.866	0.874
5. Difficulties paying for educational services or health services	0.07 (1.03)	−0.200	−0.037	0.796	0.018	−0.001	0.013	0.879	0.889
**Social activities (SA)**									
6. Participating in social gatherings	−0.26 (1.53)	0.301	−1.417	0.040	0.888	−0.018	−0.059	0.877	0.878
7. Having romantic or sexual relationships	−0.12 (1.28)	0.077	−0.895	0.046	0.786	−0.019	0.008	0.895	0.897
8. Sharing with those close to me	−0.21 (1.40)	0.215	−1.256	−0.012	0.900	−0.006	0.026	0.873	0.874
9. Physical activity	−0.21 (1.37)	0.218	−1.159	−0.031	0.759	0.054	0.036	0.897	0.899
10. Going for walks/visits	−0.24 (1.40)	0.268	−1.213	−0.013	0.860	0.052	0.023	0.873	0.876
**Habitability (H)**									
11. Having privacy in my home	−0.29 (1.14)	0.181	−0.400	−0.007	−0.049	0.899	−0.033	0.877	0.880
12. Finding silence in my home	−0.38 (1.15)	0.241	−0.502	0.049	0.043	0.921	−0.076	0.860	0.865
13. Having space to carry out my activities	−0.42 (1.12)	0.318	−0.385	0.008	0.111	0.822	−0.101	0.872	0.875
14. Cooking comfortably	−0.21 (0.98)	−0.100	−0.142	−0.015	−0.026	0.795	0.178	0.877	0.880
15. Feeling comfortable in the bathroom	−0.19 (0.94)	−0.138	0.709	−0.028	0.010	0.720	0.199	0.893	0.895
**Virtual media exposure (VME)**									
16. Using mobile applications to shop from home	0.43 (1.21)	−0.344	−0.701	0.105	−0.145	0.033	0.737	0.929	0.931
17. Being exposed to computers or television	0.68 (1.29)	−0.638	−0.600	0.005	0.040	−0.030	0.920	0.900	0.905
18. Participating in virtual meetings	0.75 (1.36)	−0.808	−0.564	−0.029	−0.004	0.041	0.867	0.911	0.915
19. Using technological devices	0.86 (1.24)	−0.819	−0.332	0.010	0.037	0.037	0.940	0.890	0.892
20. Accessing the internet	0.69 (1.23)	−0.551	−0.628	−0.003	0.018	−0.046	0.887	0.900	0.902
**Correlations**								***ω* index**	***α* index**
Economic Stressors				—	—	—	—	0.899	0.906
Social Activities				0.312^*^	—	—	—	0.904	0.906
Home Habitability				0.081	0.449^*^	—	—	0.899	0.900
Exposure to Virtual Media				0.290^*^	0.085	0.207^*^	—	0.924	0.926

^*^p < 0.001; ES, economic stressors; SA, social activities; H, home habitability; VME, exposure to virtual media; M, mean; SD, standard deviation; S, skewness standardized; K, kurtosis standardized.

Third, a multigroup CFA model was estimated between M3 men and women; the results are presented in Table 3. This model was first tested for configural invariance, i.e., a baseline model was fitted for each group separately. Compared with the configural model, the metric model showed no relevant changes in the RMSEA differential or CFI, thus confirming that the factor loadings of the items are the same in both groups. Finally, the scalar model compared with the configural model also showed no relevant changes in the RMSEA differential or CFI, which means that the intercepts of the items are the same in both groups.

**Table 3:** Fit indexes for multiple-group CFA of ECSS-20.

	*χ* ^2^	df	*χ*^2^/df	RMSEA	90% CI	CFI	TLI	SRMR	CMs	Δ CFI	ΔRMSEA
M5	610.507	328	1.861	0.074	[0.065, 0.830]	0.937	0.927	0.055		—	—
M6	628.231*	344	1.826	0.073	[0.064, 0.081]	0.936	0.930	0.057	M6-M5	−0.001	−0.001
M7	646.164*	360	1.794	0.071	[0.062, 0.080]	0.936	0.932	0.058	M7-M6	−0.001	−0.003

M5, configural invariance; M6, metric invariance; M7, scalar invariance; * = *p* < 0.001; *χ*^2^, chi-square; df, degree of freedom; RMSEA, root mean square error of approximation; 90% CI, confidence interval; CFI, comparative fit index; TLI, Tucker–Lewis index; SRMR, standardized root mean square residual; CMs, comparison between models; ΔCFI, change in comparative adjustment index; ΔRMSEA, change in the error of the mean square of the approximation root.

Thus, strong measurement invariance is demonstrated by the existence of metric and scalar invariance, i.e., the equivalence between factor loadings and thresholds for those who identified themselves as female or male is sustained (Cheung and Rensvold, 2002; Chen, 2007; Dimitrov, 2010; Leitgöb et al., 2023).

**Figure 1:**
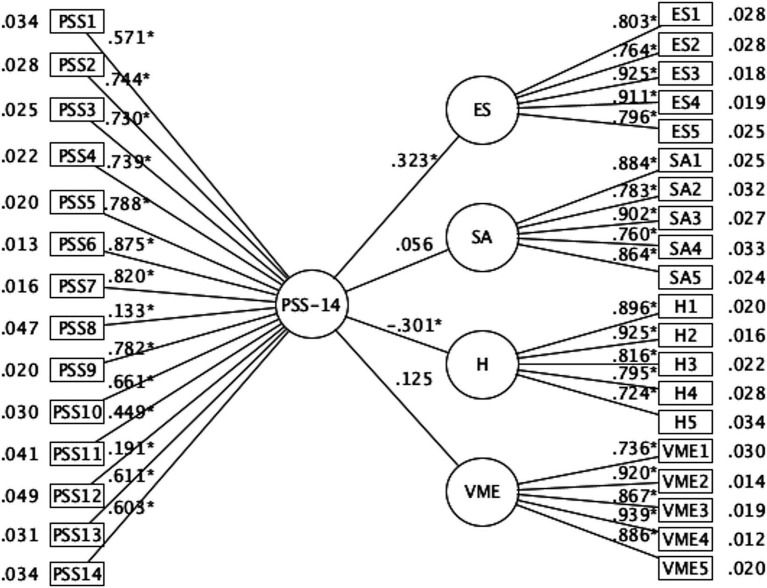
SET-ESEM ECSS-20 and PSS-14.

Finally, the SET-ESEM model that estimated the association between the latent dimensions of the ECSS-20 and the PSS-14 one-dimensional showed comparative and absolute fit indices far from the recommendations [*χ*^2^(469) = 1780.710; CFI = 0.911; TLI = 0.893; RMSEA = 0.094; 90% CI = (0.090, 0.099); SRMR = 0.094]. The observed mismatches could be attributed to the factor loadings shown by the PSS-14 (see Figure 1). Finally, significant direct and inverse loadings are observed for the ES factor (*λ* = 0.323) and the H factor (*λ* = −0.301) concerning PSS-14. The details of the standardized relationships between the latent dimensions and the PSS-14 indicators are shown in the Figure 1.

## Discussion

The present study focused on developing and validating the Environmental Stressors in Confinement Scale (ECSS-20), which assesses the perception of change in environmental stressors produced by confinement circumstances. Theoretical and practical contributions, limitations, and future lines of research emerging from this study are discussed below.

In first place, the final structure of the ECSS-20, composed of four dimensions: economic stressors (ES), social activities (SA), habitability (H), and exposure to virtual media (VME), proved to be robust and consistent with previous literature evidencing the existence of environmental stressors stemming from confinement (Ellen et al., 2021; Kunzler et al., 2021; Sheek-Hussein et al., 2021). The reason for this is that the observed factor loadings indicate that each dimension uniquely impacts the perception of environmental stress in confinement situations. McDonald’s omega (*ω* > 0.89) and Cronbach’s alpha (*α* > 0.89; Cho and Kim, 2015), internal consistency values were also shown to be satisfactory, providing evidence of the reliability of the instrument. That is, the ECSS-20 can be applied to the Chilean adult population experiencing confinement measures.

Second, the application of gender invariance tests supported the equivalence of factor loadings between women and men, suggesting that the ECSS-20 can be used in both groups without distinction. Thus, this strengthens the instrument’s usefulness, as it demonstrates that it assesses the impact of environmental stressors accurately and comparatively, as has been evidenced in other studies analyzing gender invariance in confinement contexts (Prime et al., 2021).

Third, validity tests based on the association with other variables were established by demonstrating significant relationships between the ECSS-20 and PSS-14 dimensions. Specifically, direct significant relationships were observed between the economic stressors (ES) dimension and the perception of stress. Therefore, the greater the perception of variation in the estimated household budget, the greater the feeling of stress. Likewise, inverse relationships were observed between the dimension of habitability (H) and the perception of stress. In other words, the higher the perception of well-being with those who share a dwelling, the lower the feeling of stress. As a result, this supports previous research that suggests these factors impact the change in people’s mental health (Kunzler et al., 2021; Sheek-Hussein et al., 2021). Thus, this demonstrates that habitability and economic conditions could function as protective and risk factors in the perception of stress during confinement situations, such as during the COVID-19 pandemic. Therefore, it is proposed to consider these subscales to assess these factors in confinement circumstances.

In contrast, one of the limitations of this study is the mismatch in the RMSEA values of M1, M2, and M3 in the ESEM analyses, suggesting that the model structure and accuracy of fit could be improved. This discrepancy could be explained by factors such as the complexity of the interrelationships between dimensions or the presence of variables not considered in the model (Shi et al., 2020). In addition, the sample size and the absence of validity tests on a sample other than those collected in this study should be considered as a limitation of this study. Finally, a limitation of this research is that the survey was conducted during the medium health impact phase mandated by the Chilean government, characterized by the reduction of social interactions through measures such as social distancing or confinement, including a distance of one meter between two people and the use of a permit demonstrating current vaccination status for transit in the city (Ministerio de Salud, 2022).

This context of dynamic and sometimes irregular confinements, as evidenced in studies such as Patrono et al. (2024), demonstrates the heterogeneity of the confinement experience in the country, with differentiated impacts on the mental and physical health of the population (Duarte and Jiménez-Molina, 2022; Gutiérrez-Pérez et al., 2024). The research by Dagnino et al. (2020) underscores the significant psychological impact and the high demand for psychological support in Santiago, reflecting a critical need that could influence the structure of the ECSS-20 under different confinement intensities. Governmental policies, criticized for their improvised and unequal approach, particularly in terms of gender equity (Undurraga and López-Hornickel, 2023), and the special vulnerability of minority communities (Anandarajah et al., 2024), require careful consideration in interpreting the ECSS-20 data. Recently, Rodman et al. (2024), offered a longitudinal perspective on the deterioration of youth psychopathology due to reduced socialization, a factor that must be considered when assessing the validity of the ECSS-20 in future research. Additionally, studies on the impact of the social environment, such as that by Choi et al. (2024), highlight how specific neighborhood characteristics, such as socioeconomic deprivation and disorder, can increase the risk of dementia, mediated in part by subjective loneliness. This link underscores the importance of considering how urban environments and socialization dynamics influence mental health at all life stages (Ibanez et al., 2024; Migeot et al., 2024). The ECSS-20 ability to capture variations in the perception of environmental stress could be crucial not only for a better understanding of mental health disorders in youth but also for exploring longitudinal connections with cognitive risks in later life stages, influenced by social isolation and neighborhood conditions.

Finally, future lines of research could apply the ECSS-20 in contexts where stress is generated by confinement and/or population displacement, such as those generated by natural disasters (i.e., tsunamis, fires, landslides, extreme heat, hurricanes and tornadoes) (Sandoval-Díaz and Martínez-Labrín, 2021; Birkmann et al., 2022) or by sociopolitical situations of the countries of residence (i.e., political asylum, immigration) (Kwok and Ku, 2008; Kim et al., 2021). These events, marked by critical sociopolitical dynamics and needs for rapid adaptation, present a fertile ground to assess variations in subjective well-being, coping, and fields of spatial justice and habitability (Astudillo Pizarro and Sandoval Díaz, 2019; Sandoval-Díaz et al., 2021, 2022, 2024). Moreover, employability circumstances such as job loss or absence, or significant changes in work conditions, as well as the loss of daily social activities and hospitalizations, are critical areas where the ECSS-20 could reveal significant impacts on mental and physical health (Bahamondes-Rosado et al., 2023; Pérez-Villalobos et al., 2023). It is also advisable to explore how the ECSS-20 functions across different cultures and countries, as stress and its perception can vary considerably among different environments and populations (Tasnim et al., 2024; Tonon et al., 2024). The COVID-19 pandemic, as a natural experiment, has provided a unique context to better understand these phenomena (Gormley, 2024; Ruggeri et al., 2024). Therefore, adapting and validating the ECSS-20 in diverse cultural and environmental stress contexts could enrich our understanding of the interactions between the environment, stress, and mental health, thereby broadening the practical applications of the scale in designing targeted interventions and effective public health policies.

## Conclusion

The instrument’s multidimensional structure, internal consistency, gender invariance, and evidence associated with related measures support its validity and usefulness. The ECSS-20 is a valuable tool for investigating and further understanding the effects of confinement on the population’s mental health. Future research could explore its applicability in different contexts and populations to strengthen understanding of its psychometric properties and utility in assessing confinement situations.

## Data availability statement

The raw data supporting the conclusions of this article will be made available by the authors, without undue reservation.

## Ethics statement

The studies involving humans were approved by Comité ético científico Universidad de Tarapacá. The studies were conducted in accordance with the local legislation and institutional requirements. The participants provided their written informed consent to participate in this study.

## Author contributions

JS-P: Writing – review & editing, Writing – original draft, Validation, Project administration, Methodology, Investigation, Formal analysis, Data curation, Conceptualization. RF-U: Writing – original draft, Validation, Supervision, Software, Resources, Methodology, Funding acquisition, Formal analysis, Data curation, Conceptualization. GS-P: Writing – original draft, Validation, Supervision, Project administration, Methodology, Formal analysis, Data curation, Conceptualization. JF: Writing – original draft, Investigation. KA-C: Writing – review & editing, Writing – original draft, Visualization, Validation, Project administration, Methodology, Investigation, Formal analysis, Data curation, Conceptualization.
